# Inhibition of Endoplasmic Reticulum Stress Improves Mouse Embryo Development

**DOI:** 10.1371/journal.pone.0040433

**Published:** 2012-07-13

**Authors:** Jin Yu Zhang, Yun Fei Diao, Hong Rye Kim, Dong Il Jin

**Affiliations:** Department of Animal Science & Biotechnology, Research Center for Transgenic Cloned Pigs, Chungnam National University, Daejeon, Korea; Konkuk University, Republic of Korea

## Abstract

X-box binding protein-1 (XBP-1) is an important regulator of a subset of genes during endoplasmic reticulum (ER) stress. In the current study, we analyzed endogenous XBP-1 expression and localization, with a view to determining the effects of ER stress on the developmental competency of preimplantation embryos in mice. Fluorescence staining revealed that functional XBP-1 is localized on mature oocyte spindles and abundant in the nucleus at the germinal vesicle (GV) stage. However, in preimplantation embryos, XBP-1 was solely detected in the cytoplasm at the one-cell stage. The density of XBP-1 was higher in the nucleus than the cytoplasm at the two-cell, four-cell, eight-cell, morula, and blastocyst stages. Furthermore, RT-PCR analysis confirmed active *XBP-1* mRNA splicing at all preimplantation embryo stages, except the one-cell stage. Tunicamycin (TM), an ER stress inducer used as a positive control, promoted an increase in the density of nuclear XBP-1 at the one-cell and two-cell stages. Similarly, culture medium supplemented with 25 mM sorbitol displayed a remarkable increase active XBP-1 expression in the nuclei of 1-cell and 2-cell embryos. Conversely, high concentrations of TM or sorbitol led to reduced nuclear XBP-1 density and significant ER stress-induced apoptosis. Tauroursodeoxycholic acid (TUDCA), a known inhibitor of ER stress, improved the rate of two-cell embryo development to blastocysts by attenuating the expression of active XBP-1 protein in the nucleus at the two-cell stage. Our data collectively suggest that endogenous XBP-1 plays a role in normal preimplantation embryonic development. Moreover, XBP-1 splicing is activated to generate a functional form in mouse preimplantation embryos during culture stress. TUDCA inhibits hyperosmolar-induced ER stress as well as ER stress-induced apoptosis during mouse preimplantation embryo development.

## Introduction

The endoplasmic reticulum (ER) is the site for biosynthesis of lipids, membrane proteins, and secretory proteins [1]. However, several stimuli, including mutations, chemical treatment or environmental stress, that disrupt ER homeostasis, affect proper protein folding and lead to the accumulation of unfolded and misfolded proteins in the ER lumen, followed by induction of ER stress [2]. ER stress, in turn, leads to the activation of a series of adaptive pathways known as unfolded protein response (UPR) to maintain ER homeostasis [3], [4], [5], [6]. In cases where ER stress is prolonged or too severe to resolve, apoptosis is induced [7]. In mammals, XBP-1 acts as a transcription factor to regulate downstream UPR genes [3], [8], [9], [10], [11]. During ER stress, *XBP-1* transcription is induced, and the transcript is unconventionally spliced by an endoribonuclease that removes the 26-nucleotide intron to form the spliced *XBP1* protein (*XBP-1 s*, 371 amino acids) from the premature unspliced *XBP-1* (*XBP-1 u,* 267 amino acids) form [9]. However, only *XBP-1s* can enter the nucleus and act as a functional transcription factor that up-regulates UPR target genes [3], [11]. Therefore, this specific *XBP-1* splicing variant is widely used to monitor ER stress, both *in vivo* and *in vitro* [12], [13], [14]. Signaling molecules involved in the ER stress response are required during embryonic development [10], [15], [16]. Recently, the characteristics and functions of XBP-1 were described in detail. Spliced *XBP-1* regulates a subset of ER-resident chaperone genes in the UPR and protects cells against ER stress [11], [17]. XBP-1 is widely expressed in adult tissues and essential for cell differentiation [16], [17]. The protein is required for cardiac myocyte survival [18], liver development [16], skeletal formation and terminal differentiation of immature plasma cells into immunoglobulin-secreting B-lymphocytes [5], [19], [20]. A number of studies have shown that vertebrate XBP-1 performs crucial functions during development, and is essential for the viability of embryos in both mice and frogs [16], [21]. In mice, embryonic lethality of XBP-1^−/−^ fetuses results from liver apoptosis [16]. Moreover, the *XBP-1* gene is essential for early embryonic development in *Drosophila* and Xenopus [21], [22]. Although the influence of ER stress on cellular physiology has been extensively investigated, little is known about its effects on early embryonic development in mammalian. A very recent study has reported that ER stress was present in mouse preimplantation embryos and transient activation of XBP-1 could be induced by tunicamycin (TM) in mouse embryos [23]. In the present study, we examined the effects of increased ER stress on mouse embryogenesis by treating embryos with TM, an inducer of ER stress, which prevents N-linked protein glycosylation. Simultaneously, the effects of sorbitol on preimplantation embryos were investigated. Sorbitol has been used to assess culture stress in oocytes and preimplantation embryos in earlier investigations [24], [25]. Sorbitol, a hyperosmolar stress inducer, is reported to cause an increase in apoptosis and decrease in blastocyst cell number [26]. Thus, elucidation of the mechanisms underlying the effects of culture stress on preimplantation embryo growth is critical for improving mouse embryo development *in vitro*.

To our knowledge, the expression patterns and localization of XBP-1 in mouse oocytes and preimplantation embryos and the effect of ER stress inhibition on embryo development have not been established to date. Here, we have characterized XBP-1 protein expression and localization patterns in the developmental processes of mouse preimplantation embryos, and shown that active XBP-1 could have a protective function for sorbitol hyperosmolar stress *in vitro* culture. Furthermore, tauroursodeoxycholate (TUDCA), a bile acid acting as a potent chemical chaperone that inhibits ER stress *in vitro* [27], was evaluated in terms of its ability to attenuate ER stress-induced apoptosis.

## Materials and Methods

### Chemicals

All chemicals were purchased from Sigma (St Louis, MO), unless otherwise specified.

### Preparation of Mouse Oocytes and Embryos

All animal procedures were approved by the Institutional Animal Care and Use Committee of Chungnam National University. Oocytes at the germinal vesicle stage were obtained from ICR female mice (Charles River) as cumulus-oocyte complexes (COCs). Five- to seven-week old females were induced to superovulate via injection with 5 IU PMSG (Sigma) and sacrificed 48 h later. Ovaries were recovered in FHM medium (Millipore). COCs were mechanically removed, and oocytes washed by pipetting in FHM containing 0.1% (w/v) hyaluronidase (Sigma). Oocytes containing germinal vesicles were collected. Mature MII oocytes were collected as COCs after PMSG injection, followed by injection of 5 IU hCG (Sigma) after 44 h. Mice were killed at 18 h after hCG (hphCG) injection. COCs were removed from oviducts into FHM, following which oocytes were denuded using hyaluronidase and collected for experiments. To obtain zygotes and embryos, female mice were coupled with males after hCG injection, and killed 18, 44, 64, 72, 88 and 96 hphCG to recover one-cell, two-cell, four-cell, eight-cell embryos, morulae, blastocysts, respectively.

### Culture and Treatment of Embryos *in vitro*

To determine the effects of TM, sorbitol or TUDCA on embryo development, two-cell stage embryos were recovered at 44 h phCG, and cultured without or with TM, sorbitol or TUDCA. The required drug concentrations were prepared from stock solution diluted in M16. Groups of 25–30 embryos were placed in warmed 40 µL droplets of culture medium, covered with mineral oil, and cultured with 5% (v/v) CO_2_ at 37°C.

### Reverse Transcription-polymerase Chain Reaction

To analyze gene expression, mature oocytes at various stages and early embryonic stages were collected. Total mRNA was extracted using RNeasy® Mini Kits (Qiagen, Valencia, CA), according to the manufacturer’s instructions. For reverse transcription, total mRNA in a final volume of 20 µL (containing 0.5 mg oligo-dT, RT buffer [1×], 10 mM dithiothreitol, and 10 mM dNTP) was subjected to reverse transcription at 37°C for 50 min, followed by 70°C for 15 min, and products were stored at 4°C until use. Each RT-PCR reaction mixture was composed of 4 µL cDNA and 10 pm/µl of the appropriate forward and reverse primers (Table 1). Tests were performed in triplicate, and the mRNA level in each sample normalized to that of β-actin mRNA.

**Table 1:** All primer sequences used in the experiments.

Gene	Primer	Sequence	Accession number	Annealing (°C )	Product size(bp)	
*XBP-1*	Forward Reverse	ACTCGGTCTGGAAATCTG TAGCCAGGAAACGTCTAC	AF027963	60		279
*β-actin*	Forward Reverse	ATATCGCTGCGCTGGTCGTC AGGATGGCGTGAGGGAGAGC	NM-007393	60		517

### Immunofluorescence Analysis

Mouse embryos at various stages were fixed in 4% (v/v) paraformaldehyde for 30 min at room temperature, and permeabilized with 0.1% (v/v) Triton-100 for 30 min. Oocytes and embryos were blocked overnight with 3% (w/v) BSA in PBS at 4°C, and subsequently incubated with rabbit polyclonal anti-XBP-1 antibody (Santa Cruz Biochemicals, Santa Cruz, CA) diluted in blocking solution for 1 h at 37°C. The anti-XBP-1 antibody was produced from the epitope corresponding to amino acids 76–263 of mouse XBP-1. After washing with 0.5% (v/v) Tween-20 in PBS, samples were reacted with anti-rabbit FITC-conjugated secondary antibody in blocking solution for 60 min at 37°C. Next, samples of various developmental stages were mounted using VECTASHIELD® Mounting Medium containing DAPI. Images were obtained using a Zeiss scanning laser confocal microscope, and analyzed with LSM Image Browser software. At least 20 oocytes or embryos were examined for every stage.

### Terminal Deoxynucleotidyl Transferase dUTP Nick End Labeling (TUNEL) Assay

Blastocysts were washed three times in PBS (pH 7.4) containing polyvinylpyrrolidone (PVP; 1 mg/mL) followed by fixation in 4% (v/v) paraformaldehyde in PBS for 1 h at room temperature (RT). After fixation, parthenotes were washed in PVA-PBS and permeabilized by incubation in 0.3% (v/v) Triton X-100 for 1 h at RT. Embryos were washed twice in PVA-PBS, and incubated with fluorescein-conjugated dUTP and terminal deoxynucleotidyl transferase (provided in the *in situ* Cell Death Detection Kit; Roche; Mannheim, Germany) in the dark for 1 h at 37°C. After counterstaining with 40 µg/mL propidium iodide (PI) and 50 µg/mL RNaseA for 1 h at 37°C to label all nuclei, embryos were mounted with slight coverslip compression and observed under a confocal microscope.

### Western Blotting

Mouse embryos at various stages (100 per sample) were washed three times in PVA-PBS, and resuspended in extraction buffer (PRO-PREP; Intron Biotechnology, Seong, Korea). Extracted proteins were separated by 10% (w/v) SDS-PAGE using Bio-Rad apparatus (Bio-Rad, Hercules, CA) and electrophoretically transferred to membranes using a Bio-Rad Mini Trans-Blot Cell. Membranes were blocked with 5% (w/v) skimmed milk and 0.5% (v/v) Tween-20 in Tris-buffer saline and subsequently exposed to primary antibodies directed against XBP-1 and β-actin (Santa Cruz Biochemicals) dissolved in Tris-buffered saline containing 5% (w/v) non-fat dry milk powder and 0.1% (v/v) Tween-20. Membranes were washed in Tris-buffered saline with 0.5% (v/v) Tween-20 for 15 min, and antibody–antigen complexes detected using anti-mouse IgG or anti-rabbitIgG peroxidase conjugates, followed by application of an ECL detection kit (AmershamBioscience, Piscataway, NJ). All experiments were performed in triplicate. The intensities of bands on the blots were measured densitometrically (Bio-Rad). The β-actin band served as a control.

### Statistical Analysis

All data were analyzed using one-way ANOVA and Fisher’s protected least significant difference (LSD) test with general linear models of the Statistical Analysis System (SAS, Cary, NC) program to determine the differences among experimental groups. Treatment differences were considered significant at *P* values<0.05.

## Results

### Localization of XBP-1 in Mouse Oocytes and Preimplantation Embryos

**Figure 1:**
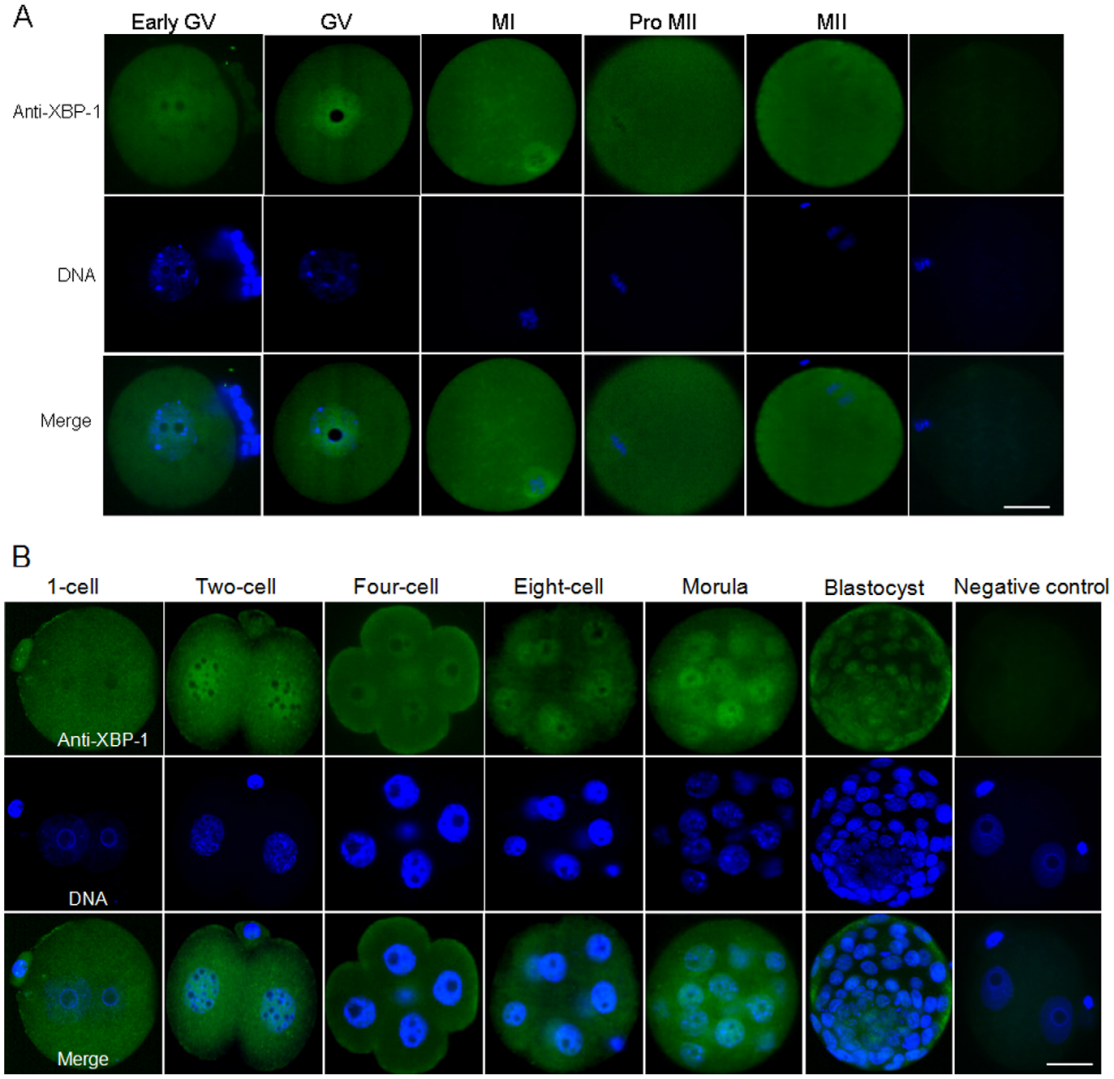
Detection of endogenous XBP-1 in mouse oocytes and preimplantation embryos *in vivo*. A. A specific anti-XBP-1 antibody was used to detect localization of XBP-1 in mouse oocytes via immunostaining (green). Nuclei were stained with DAPI (blue); Scale bar, 20 µm. B. Confocal immunofluorescence images of mouse pre-implantation embryos. The XBP-1 protein was detected using a specific antibody (green). Negative control embryos were probed directly with the secondary antibody. Nuclei were stained with DAPI (blue); Scale bar, 20 μm.

To determine whether the ER stress signaling pathway is essential for maturation of mouse oocytes and development of pre-implantation embryos, XBP-1 was used as a marker, as described previously [28]. We initially examined the localization of XBP-1 protein in mouse mature oocytes and pre-implantation stage embryos via immunostaining using a specific anti-XBP-1 antibody. Mouse XBP-1 was localized principally in the nuclei and weakly in the cytoplasm at the GV, two-cell, four-cell, eight-cell, morula and blastocyst stages (Fig. 1B). In contrast, XBP-1 was mainly detected in the cytoplasm at the one-cell stage. During the meiotic stages, XBP-1 localized to the spindle microtubules in metaphase I oocytes, but the XBP-1 signal at the spindle microtubules weakened progressively in pro-metaphase II and metaphase II oocytes (Fig. 1A).

### Characterization of Mouse *XBP-1 *mRNA Splicing in Early Stage Embryos

**Figure 2:**
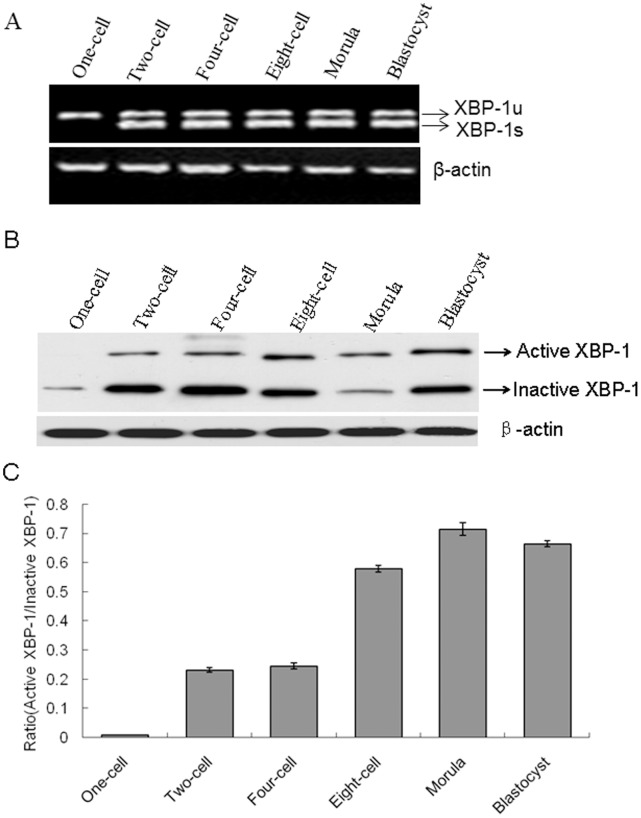
Detection of *XBP-1* splicing in mouse preimplantation embryos. A. Expression of *XBP-1* mRNA was analyzed using RT-PCR. RNA was isolated from 50 embryos of each stage and reverse-transcribed. cDNA was used as the template for PCR. *XBP-1 *s and *XBP-1 u* amplicons were separated on a 2% (w/v) agarose gel. B. Expression patterns of active and inactive XBP-1 proteins were detected in two-cell embryos using Western blotting. β-actin served as the control. C. Quantification of the Western blot analysis in B. The data were presented as means ± SD from three independent experiments.

*XBP-1* mRNA is spliced into *XBP-1s* and *XBP-1u* forms. However, only the XBP-1s form creates a translational frame shift and is functionally active in the nucleus [11], [29]. Using RT-PCR analysis, both *XBP-1s* and *XBP-1u *mRNAs were clearly detected at the two-cell, four-cell, morula and blastocyst stages, while the *XBP-1u* transcript was solely identified at the one-cell stage (Fig. 2A). Consistently, Western blotting analysis disclosed the presence of both activated XBP-1 from *XBP-1s* mRNA and inactivated XBP-1 from *XBP-1u* mRNA proteins at the two-cell, four-cell, morula and blastocyst stages (Fig. 2B), and only the inactivated XBP-1 protein at the one-cell stage. Our findings indicate that XBP-1 plays a role in the development of mouse preimplantation embryos.

### Effects of Culture Stress on Mouse Preimplantation Embryo Development *in vitro*

**Figure 3:**
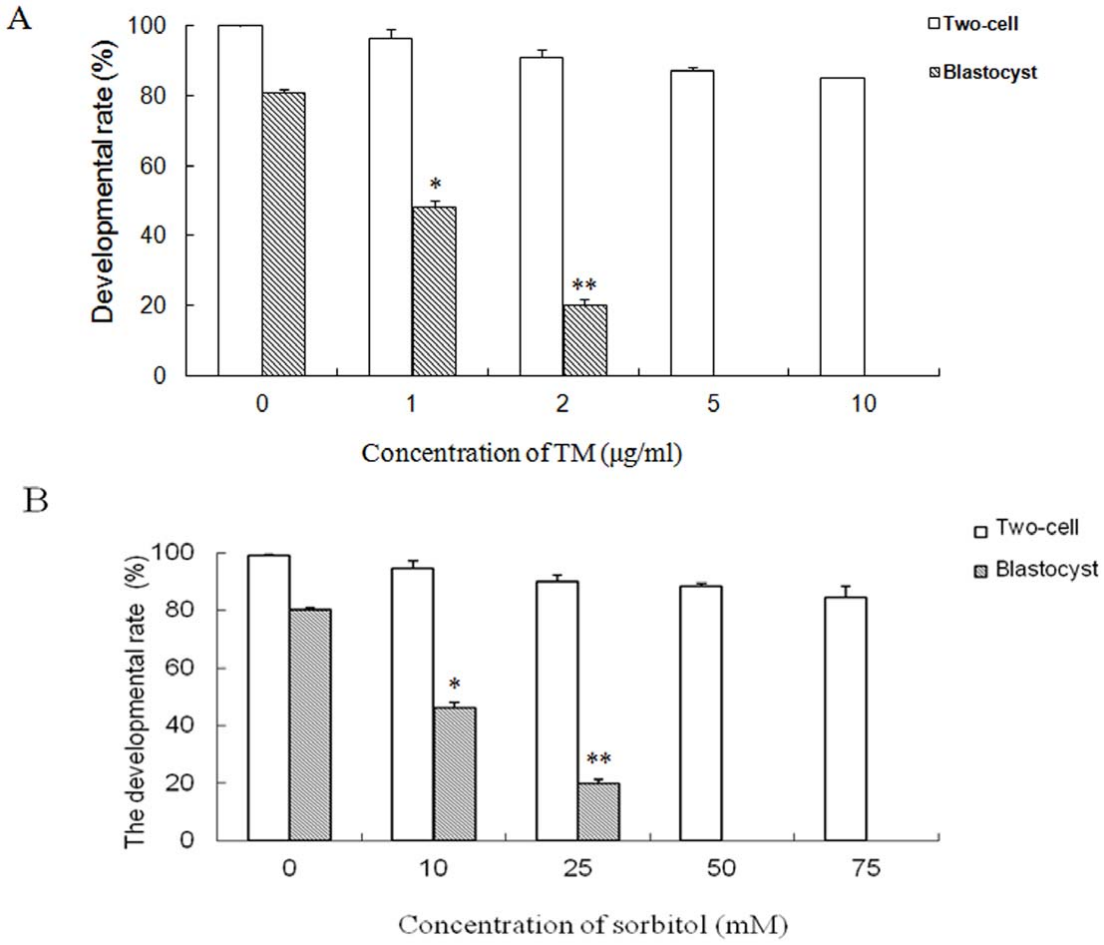
Effects of stress inducers on mouse embryo development *in vitro*. A. Two-cell embryos collected at 44 h phCG were cultured in M16 containing 0, 1, 2, 5 and 10 μg/ml TM, for 60 h. B. Two-cell embryos were cultured in M16 containing 0, 10, 25, 50, 75 mM sorbitol, for 60 h. (*) indicates a statistically significant difference (P<0.05) and (**) indicates a statistically significant difference from control (P<0.01).

Tunicamycin (TM), a compound that inhibits N-linked glycosylation in newly synthesized polypeptides, induces ER stress [30]. Sorbitol is usually employed to induce osmotic stress in embryonic development *in vitro*. To evaluate the effects of culture stress on rate of blastocyst development, one-cell stage embryos were cultured in M16 supplemented with varying concentrations of TM or sorbitol. One-cell stage embryos treated with 2 μg/ml TM or 25 mM sorbitol displayed a significant decrease in the blastocyst developmental rate (Fig. 3). However, in the absence of drugs, about 80% of two-cell stage embryos reached the blastocyst stage after 60 h. Embryos treated with more than 5 μg/ml TM or 50 mM sorbitol were completely blocked at the two-cell stage, and did not develop into blastocysts. The results indicate that prolonged culture stress results in arrest at the two-cell stage.

### Induction of Active XBP-1 in the Nucleus of One-cell Stage Embryos by Stress Inducers

**Figure 4:**
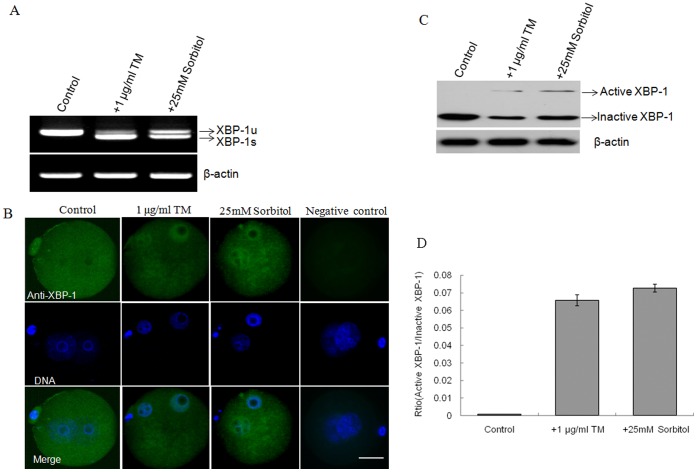
Induction of active XBP-1 in one-cell stage embryos. A. *XBP-1* mRNA was spliced to produce the spliced and unspliced forms in the presence of TM and sorbitol. B. Immunofluorescence micrographs of two-cell stage embryos with TM or sorbitol. Active XBP-1 protein was detected in nuclei in the presence or absence of stress inducers (green). Negative control embryos were probed directly with the secondary antibody. Nuclei were stained with DAPI (blue). Scale bar, 20 μm. C. Active and inactive XBP-1 proteins were detected in the presence and absence of stress inducers using Western blotting. β-actin served as a control. D. Quantification of the Western blot analysis in C. The data were presented as means ± SD from three independent experiments.

To explore the possible relationship between culture stress and ER stress, one-cell stage mouse embryos were cultured respectively with or without TM and sorbitol. RT-PCR analysis showed that the spliced *XBP-1* form was absent in normal one-cell stage embryos, but present in the embryos treated with 1 μg/ml TM or 25 mM sorbitol (Fig. 4A). Consistent with RT-PCR results, Western blotting revealed the presence of active and inactive XBP-1 proteins in one-cell embryos treated with 1 μg/ml TM or 25 mM sorbitol. In contrast, only inactive XBP-1 protein was detected in normal one-cell stage embryos (Fig. 4C). In addition, immunostaining analysis disclosed localization of XBP-1 protein specifically in the cytoplasm of one-cell stage embryos that were not treated with TM or sorbitol, but in both the nucleus and cytoplasm of embryos exposed to TM or sorbitol (Fig. 4B). Based on the data, we propose that 1 μg/ml TM and 25 mM sorbitol represent the optimum doses to induce ER stress in early stage embryos.

### Effect of Stress Inducers on XBP-1 Protein in Two-cell Stage Embryos

**Figure 5:**
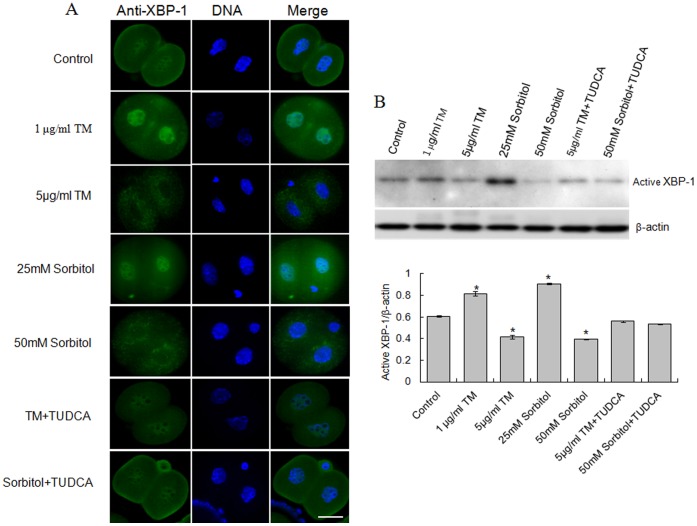
Dose-dependent effects of stress inducers on localization of XBP-1. A. Two-cell stage embryos cultured with activator or inhibitor of ER stress were examined using a specific anti-XBP-1 antibody (green). Nuclei were stained with DAPI (blue). Scale bar, 20 μm. B. Active XBP-1 in two-cell embryos in the presence of activator or inhibitor of ER stress was analyzed using Western blotting. β-actin served as the control. Each experiment was repeated three times. (*) indicates a statistically significant difference from control (P<0.05).

To investigate the mechanism by which stress inducers block the development of embryos, two-cell embryos were cultured in the absence or presence of different concentrations of TM and sorbitol, respectively. Notably, XBP-1 protein was localized in the nucleus of two-cell stage embryos in the absence of stress inducers, as shown in Fig. 5A, with a weak signal detected around the cytoplasm. As expected, after 1 μg/ml TM or 25 mM sorbitol treatment for 3 h in culture medium, localization of XBP-1 protein to the nucleus was significantly increased. We also observed the XBP-1 protein in the cytoplasm, but not the nucleus, when two-cell stage embryos were treated with higher concentrations of stress inducers (5 μg/ml TM or 50 mM sorbitol) for 3 h. Interestingly, after treatment with higher concentrations of stress inducers plus TUDCA, a chemical chaperone that acts as an ER stress inhibitor, XBP-1 was re-localized to the nucleus at the two-cell stage embryo. In Western blotting analysis, the expression pattern of activated XBP-1 protein (XBP-1s) in two-cell stage embryos treated with ER stress inducers or inhibitors was consistent with immunostaining data (Fig. 5B). These results suggest that nuclear XBP-1 may be functional, but not cytoplasmic XBP-1.

### Effect of TUDCA on Mouse Embryo Development

To ascertain whether TUDCA attenuates ER stress induced-apoptosis in mouse preimplantation embryos, we cultured one-cell stage embryos supplemented with TM as a positive control for induction of ER stress and sorbitol as an inducer of hyperosmolar stress. The rates of blastocyst formation observed in the presence of TM or sorbitol alone were significantly lower than those of control group (43.7±3.2%, 20.0±1.4% *vs*. 81.1±0.7%, respectively). However, the embryo cleavage rates (87.0±0.6%, 88.7±0.8% *vs.* 87.1±2.4%, respectively) and development rate to blastocysts in cultures treated with TM plus TUDCA or sorbitol plus TUDCA were significantly similar to that of control (80.3±1.5%, 79.8±2.6% *vs.* 81.1±0.7%, respectively) (*p*<0.05) (Table 2). Moreover, TUDCA induced not only a remarkable improvement in cleavage and developmental rates into blastocyst, compared with control (94.9±1.8%, 95.8±0.8% *vs.*87.1±2.4% *and* 81.1±0.7%, respectively), but also a significant increase in the total cell number of blastocysts (*p*<0.05).

**Table 2:** Effects of culture stress on mouse embryonic development *in vitro*.

Group	No. of oocyte	Cleavage (%)	% Blastocyst (n)	Mean (±SD) no. of total nuclei
Control	201	87.1±2.4^a^	81.1±0.7^a^ (142)	104.3±2.1^a^
TM	197	88.4±1.4^a^	43.7±3.2^b^ (76)	95.7±1.5^b^
Sorbitol	197	86.3±1.3^a^	20.0±1.4^c^ (34)	91.3±1.2^b^
TUDCA	198	94.9±1.8^b^	95.8±0.8^d^ (180)	119.3±1.2^c^
TM+TUDCA	192	87.0±0.6^a^	80.3±1.5^a^ (134)	101.3±0.6^a^
Sorbitol+TUDCA	195	88.7±0.8^a^	79.8±2.6^a^ (138)	103.0±1.0^a^

In the same column, values with different superscripts (a, b, c, d) indicate significantly different numbers (P<0.05).

### Effects of Stress Inducers on Apoptosis in Blastocysts

**Figure 6:**
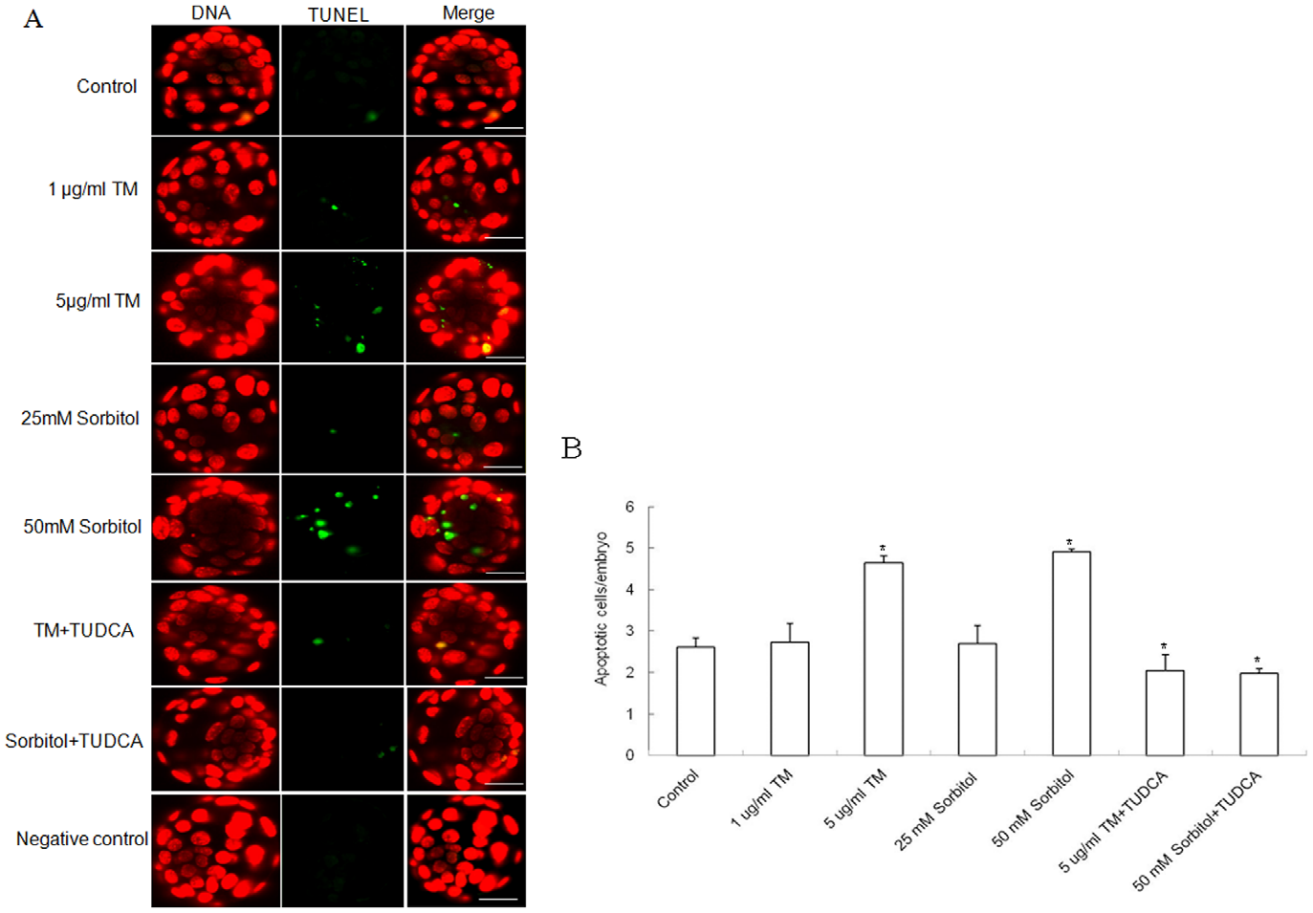
Effect of TUDCA on apoptosis in mouse blastocyst. Apoptosis in blastocysts was evaluated using the TUNEL assay. The original magnification was approximately × 200. A. Images of mouse blastocysts cultured with stress inducers in the presence or absence of TUDCA. The green color indicates DNA fragmentation, and nuclei are stained in red; Scale bar, 20 μm. B. Mean values ± SEM of number of DNA fragments in blastocysts cultured with stress inducers in the presence or absence of TUDCA. Each experiment was repeated three times. (*) indicates a statistically significant difference from control (P<0.05).

The TUNEL assay was used to evaluate the quality and viability of mouse blastocysts grown in cultures supplemented with stress inducers [31], [32]. In our experiments, the calculated percent apoptosis was not significantly different between blastocysts treated with 1 μg/ml TM or 25 mM sorbitol and the control group. However, DNA fragmentation in blastocysts treated with higher concentrations of stress inducers, i.e., 5 μg/ml TM (n = 21) or 50 mM sorbitol (n = 18), was significantly increased, compared with the control group (n = 25). Supplementation of the medium with TUDCA (TM+TUDCA, n = 18; sorbitol+TUDCA, n = 20) in addition to culture stress inducers led to a decrease in the number of apoptotic blastocysts, similar to that observed in the control group and in the presence of low doses of culture stress agents (1 μg/ml TM, n = 22; 25 mM sorbitol, n = 19) (Figure 6). Our findings clearly support the theory that TUDCA attenuates ER stress-induced apoptosis during mouse embryonic development.

## Discussion

XBP-1 regulates a subset of endoplasmic reticulum resident chaperone genes in the unfolded protein response in mammalian cells [11], [33]. In mice, complete XBP-1 deficiency results in embryonic lethality [16]. An endogenous ER stress indicator has been designed to monitor ER stress during development, tissue maturation and aging *in vivo* [12]. These earlier studies collectively demonstrate that XBP-1 is an essential transcription factor which regulates target genes or regulator during ER stress. The XBP-1 gene appears to be essential in embryogenesis in *Drosophila* and mouse [12], [34]. However, to date, few reports have documented the function of XBP-1 during the development of mouse preimplantation embryos *in vitro* [23]. In the current study, we initially examined the localization of endogenous XBP-1 protein in unstressed oocytes and preimplantation embryos *in vivo*. XBP-1 was mainly localized in the nucleus of GV oocytes and spindle microtubules in metaphase I oocytes, but the signal weakened progressively in pro-metaphase II and metaphase II oocytes. This result is slightly different from our previous data on XBP-1 localization in porcine oocytes *in vitro* [35]. In addition, endogenous XBP-1 protein was predominantly localized in the nucleus, and weakly but uniformly distributed throughout the cytoplasm from the two-cell to blastocyst stages. Interestingly, only cytoplasmic expression of XBP-1 was detected at the one-cell stage (Figure 5). Active XBP-1 is known to translocate into the nucleus to adapt the ER stress response [36]. Therefore, the nuclear localizations of XBP-1 in different stages of embryos indicate that there exists ER stress in preimplantation embryo development. Two-cell stage in mouse embryos is the time of major embryonic genome activation. After fertilization, a major level of transcription is not observed until embryonic genome is activated [37]. Thus, we believe that extensive nuclear accumulation of XBP-1 at the two-cell stage appears to be significantly associated with the major step of embryonic genome activation. To characterize XBP-1 splicing, we designed a specific primer to detect *XBP-1* mRNA in preimplantation embryos. The protein expression pattern of XBP-1 was consistent with the *XBP-*1 transcript level, signifying that XBP-1 splicing is associated with the development of mouse preimplantation embryos. Our findings are also in keeping with data obtained on XBP1-EGFP splicing in earlier stage Drosophila larvae indicating that occasional *XBP-1* splicing occurs during normal development [38]. Although the *in vivo* function of XBP-1 has not been precisely analyzed, its high expression suggests a conserved function in mouse preimplantation embryos. There were some evidences for the relation between ER stress and embryo development. In previous studies, XBP-1 was shown to be essential for mouse placental development [39]. HSPA5 (GRP78/BiP), a central regulator for ER stress, was required to be ensure early mouse embryo development [40]. Additionally, the UPR contributed to pre-implantation mouse embryo death when the DDK syndrome (embryonic lethal phenotype in crosses involving the DDK inbred strain) was evident [41]. These suggest that ER stress plays a critical role for embryo development.

Different complex formulations of culture media may induce stress via distinct mechanisms [42]. Stress-activated proteins have been thought to be necessary for preimplantation embryonic development [43]. Several cellular stresses including oxidative stress, heat-shock stress or culture stress have been widely demonstrated in *in vitro* embryo development [44], [45], [46]. In the current study, we used sorbitol as the stressor and analyzed XBP-1 splicing in the development of preimplantation embryos under conditions of hyperosmolar stress. TM, a chemical reagent, is generally used to induce ER stress [35]. Accordingly, we used TM as a positive control to induce *XBP-1* mRNA splicing for examining the effects of ER stress on the development of mouse preimplantation embryos. When two-cell stage embryos were cultured with low doses of stressors (1 μg/ml TM or 25 mM sorbitol), the development rate of blastocysts was significantly decreased. Low doses of TM or sorbitol induced not only *XBP-1* mRNA splicing but also expression of active XBP-1 protein, and relocalization of the protein from the cytoplasm to the nucleus in embryos at the one-cell stage (Fig. 4A). These results indicate that sorbitol induces XBP-1 splicing and mild ER stress, similar to other known ER stress inducers [47]. Our findings were consistent with previous studies using human HEK293 cells and embryonic stem cells, XBP-1 splicing decreased and even disappeared with prolonged ER stress [48], [49]. Spliced XBP-1 mRNA was reported in preimplantation embryos with response to TM treatment in mice [23]. Our studies further demonstrated the localization of active XBP-1 in nucleus of mouse preimplantation embryos. To our knowledge, this is one of the first observations of increased expression of XBP-1 at both the mRNA and protein levels in response to osmotic stress in mouse preimplantation embryos. Although osmotic shock (sorbitol) induces a heat shock protein response involving molecular chaperones that bind to unfolded and denatured proteins in normal human keratinocytes [50], [51], [52], there are no reports on induction of ER stress by sorbitol. Surprisingly, in the presence of high concentrations of TM or sorbitol, development of all two-cell stage embryos to blastocysts was blocked, as evident from the finding that XBP-1 failed to enter the nucleus (Figure 5A). Complete absence of nuclear XBP-1 in treated 2-cell embryos may be attributable to exposure to acute stress. These data clearly imply that nuclear translocation of XBP-1 and subsequent functional activities are essential for development of the mouse embryo. Also, studying the molecular mechanism of culture stress on mouse embryos could be unveiling new insights into human and other animal embryo development *in vitro* culture.

Chemical chaperones, such TUDCA, represent a group of compounds that modulate ER function, protecting against UPR induction and ER stress-induced apoptosis [31], [41], [42]. Treatment with TUDCA attenuated acute ER stress induced by TM or sorbitol and recovered localization of active XBP-1 protein to the nucleus. Under ER stress conditions, XBP-1 mRNA is processed by unconventional splicing and translated into a functional transcription factor in the nucleus [33]. We believe that the development rate of blastocysts is suppressed during acute stress conditions, since TUDCA attenuated XBP-1 activity in the nucleus of embryos at the two-cell stage. As shown in Fig. 5, the amount of active XBP-1 protein in the nucleus was significantly decreased, so that the development of two-cell stage embryos was completely blocked. In addition, TUDCA functions as a chemical chaperone, and can prevent apoptosis by blocking an ER stress-mediated apoptosis pathway in mammals [53], [54]. Our results support the theory that TUDCA assists in embryonic development and abolishes DNA fragmentation, an apoptotic signal, in blastocysts. Thus, TUDCA not only affords protection against ER stress but also contributes to the development of mouse preimplantation embryos.

In conclusion, endogenous XBP-1 appears to play an essential evolutionary role in embryogenesis. Our data provide new insights into the mechanisms underlying ER stress-mediated in embryonic development and the possible role of the anti-apoptotic effect of TUDCA on the improvement and recovery of pre-implantation embryos *in vitro* from culture stresses. Furthermore, it might have a benefit for improving embryonic development during *in vitro* culture.
